# The *Veiled Virgin* illustrates visual segmentation of shape by cause

**DOI:** 10.1073/pnas.1917565117

**Published:** 2020-05-15

**Authors:** Flip Phillips, Roland W. Fleming

**Affiliations:** ^a^Department of Motion Picture Science, Rochester Institute of Technology, Rochester, NY 14623-5608;; ^b^Department of Psychology and Neuroscience, Skidmore College, Saratoga Springs, NY 12866;; ^c^Department of Psychology, Justus-Liebig-University Giessen, 35394 Giessen, Germany;; ^d^Center for Mind, Brain and Behavior, Marburg University, 35037 Marburg, Germany;; ^e^Center for Mind, Brain and Behavior, Giessen University, 35394 Giessen, Germany

**Keywords:** perception, vision, art, transparency, perceptual organization

## Abstract

Three-dimensional (3D) shape perception is one of the most important functions of vision. It is crucial for many tasks, from object recognition to tool use, and yet how the brain represents shape remains poorly understood. Most theories focus on purely geometrical computations (e.g., estimating depths, curvatures, symmetries). Here, however, we find that shape perception also involves sophisticated inferences that parse shapes into features with distinct causal origins. Inspired by marble sculptures such as Strazza’s *The Veiled Virgin* (1850), which vividly depict figures swathed in cloth, we created composite shapes by wrapping unfamiliar forms in textile, so that the observable surface relief was the result of complex interactions between the underlying object and overlying fabric. Making sense of such structures requires segmenting the shape based on their causes, to distinguish whether lumps and ridges are due to the shrouded object or to the ripples and folds of the overlying cloth. Three-dimensional scans of the objects with and without the textile provided ground-truth measures of the true physical surface reliefs, against which observers’ judgments could be compared. In a virtual painting task, participants indicated which surface ridges appeared to be caused by the hidden object and which were due to the drapery. In another experiment, participants indicated the perceived depth profile of both surface layers. Their responses reveal that they can robustly distinguish features belonging to the textile from those due to the underlying object. Together, these findings reveal the operation of visual shape-segmentation processes that parse shapes based on their causal origin.

## Significance

How the brain reconstructs three-dimensional object shape from two-dimensional retinal light patterns remains a mystery. Most research has investigated how cues—such as shading, texture, or perspective—help us estimate visible surface points on the outside of objects. However, our findings show the brain achieves much more than this. Observers not only infer the visible outer surface but also the hidden internal structure of objects—seeing “beneath the skin.” Our findings suggest the brain parses shapes’ features according to their physical causes, potentially allowing us to separate a single continuous surface into multiple superimposed depth layers. This ability likely aids our interactions with objects, by indicating which surface locations are firmly supported from the inside and thus suitable for grasping.

**Fig. 1.**
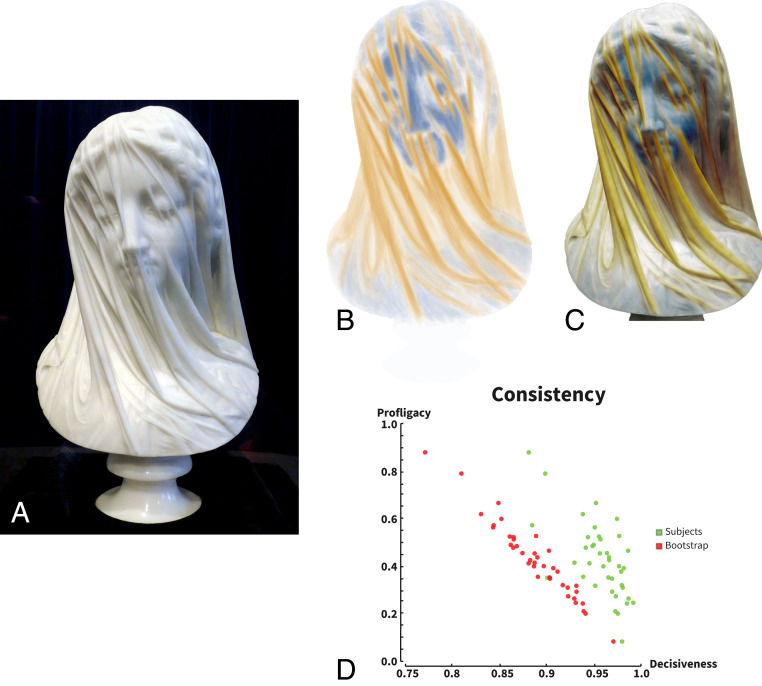
(*A*) Strazza’s sculpture *The Veiled Virgin* (approximately 1850), which elicits a vivid impression of a face “seen through” an overlying diaphanous veil (image: Wanita Bates, Presentation Archives). (*B*) Experiment 1: mean responses from 40 participants for one stimulus from the virtual painting task. Blue indicates contact responses; yellow indicates fabric responses (see *SI Appendix*, Fig. S1 for all stimuli). (*C*) The same data superimposed on stimulus image. (*D*) Profligacy and decisiveness of participants responses (see *Materials and Methods* for definitions). Green indicates mean responses for individual participants. Red indicates bootstrapped measurements for each participant.

In the 19th century, a movement emerged in sculpture that prized the depiction of figures whelmed or draped in different kinds of cloth. A canonical example of such work is Giovanni Strazza’s *The Veiled Virgin* (approximately 1850; Fig. 1). Hewn from a solid block of Carrara marble, the sculpture vividly depicts the Virgin’s face beneath a diaphanous veil. From the point of view of artistic craftsmanship, such works allowed the sculptor to display their virtuosity by subtly rendering different material properties. For example, in this case, the delicate purity of the Madonna’s complexion is enhanced by juxtaposing it with the immaculate, almost weightless transparent textile. However, from a scientific point of view, such sculptures are not only beautiful; they also raise profound questions about the nature of visual representations of surface shape and material properties (1, 2). As the sculpture consists of a single lump of solid, homogeneous material, how is it that we perceive multiple superimposed surfaces, with distinct shapes and material properties? Every visible point on the surface is just marble, and yet, somehow, we perceive one shape behind, or inside another, effortlessly distinguishing the shape features that belong to the Virgin’s face from those that belong to the overlying fabric. This suggests that the visual system may decompose the observed, composite shape into multiple “casual layers” (3–5), a process that we call “shape scission” (6, 7). At the very least, we seem to be able to identify that different three-dimensional (3D) shape features have distinct causal history, as has been suggested previously for two-dimensional (2D) shapes (8–16). We sought to measure and understand this phenomenon.

## Results and Discussion

### Experiment 1: High Intersubject Agreement about Causation in *The Veiled Virgin* Sculpture.

We reasoned that if the visual system can segment shape features based on their causal origin, participants should be able to indicate the apparent causes of different structures in the sculpture. To test this, we presented 40 untrained observers with images of *The Veiled Virgin* on a tablet device and asked them to paint onto the picture to indicate geometrical features caused predominantly by the underlying face (“contact”) and those caused predominantly by the overlying textile (“fabric”). Participants could toggle back and forth between two separate screens, one for each of the two phenomenological “layers.” The mean responses across participants for one such stimulus are shown in Fig. 1 (see *SI Appendix*, Fig. S1 for all stimuli). Blue areas denote regions indicated as being in contact with the underlying surface. Orange areas indicate regions indicated as being above the underlying surface. The responses show that, on average, participants distinguished very clearly between the two types of features; visual inspection suggests that participants very carefully teased features apart depending on their causal origin.

As a more formal test of the internal consistency of participants’ settings across the two causes, we devised two measures: “profligacy,” describing participants’ willingness to mark pixels in either of the two maps; and “decisiveness,” which measures the extent to which markings in the two maps were mutually exclusive (see *Materials and Methods* for definitions). We reasoned that if participants distinguish between causes, then pixels that are labeled as being due to one cause should not also be labeled as being due to the other. For example, if a ridge is due to the nose (contact), it cannot also be due to the textile rippling under its own self-organizing internal properties (fabric). Fig. 1*D* summarizes the distribution of profligacy and decisiveness across all markings from all participants.

It is useful to consider the intimately interconnected nature of these measures, as revealed by the pronounced negative relationship between them. When there are very few markings (i.e., low profligacy), the two sets of markings will tend not to overlap—even if generated at random—yielding high values of decisiveness. To appreciate this, consider the extreme case where participants indicate just a single pixel per layer: the probability of overlap is negligible. In contrast, if participants marked more than half the pixels in both layers, overlap would be inevitable, yielding lower decisiveness values.

Thus, to test whether participants’ settings were more decisive than would be expected by chance given the total number of markings they made, we ran a bootstrapping simulation with 10,000 repetitions, in which random (white noise) markings were created independently for pairs of contact and fabrics maps, based on the participants’ profligacy. For each simulated pair of maps, we computed the resulting decisiveness. The bootstrapping simulation was performed with the marking responses of each observer independently, facilitating within-observer comparison between the simulation and the actual responses. This analysis allows us to characterize decisiveness across participants that ranged substantially in how parsimonious or profligate they were with their markings. A within-subject paired comparison *t* test reveals that observers’ distributions are significantly more decisive than their corresponding simulations—36 of 40 participants showed significant differences between simulated and actual markings (*P* < 0.05), and 34 of 40 were significant with *P* < 0.01. This indicates that participants are highly internally consistent when distinguishing the causal origin of features in the statue.

### Experiment 2: Causal Assignment of Features on Unfamiliar Objects.

The responses to *The Veiled Virgin* sculpture provide a first hint that observers can visually distinguish the causal origin of geometrical features. However, there are a number of important limitations with the use of such sculptures to investigate causal segmentation. First, because the underlying shape is a face, some of the observers’ success may be due to recognizing familiar features such as the nose, cheeks, or eyes, rather than a more general-purpose segmentation process. Second, we do not know what the true shape of the two constituent layers is, so we can only compare participants’ responses with one another and not with the “ground truth.” Third, careful inspection of the sculpture, and our attempts to recreate similar shapes by draping diaphanous tissues over real people’s faces, revealed that the shapes carved by the sculptor are not a faithful reproduction of the geometry of the draped textile. Instead, to convey the textile as transparent, the artist created a very specific type of composite that is quite unlike the geometry that results from real drapery. Specifically, his depiction alternates over space between rendering the shape of the overlying surface, as observed directly, and the underlying face as if it were in plain view (i.e., unobscured by the cloth). This can be seen clearly, for example, in the eye, which is rendered as if unobscured almost everywhere but with narrow ridges depicting the drapery. (See stimulus 4 in *SI Appendix*, Fig. S1 as an example.) Undoubtedly, great skill was required to make these selections and to execute the transitions between them in such a way as to elicit the impression of a cloth extending continuously over the face, rather than a patchy appearance of a cloth with holes in it, or a face with stalactite-like accretions. Indeed, understanding how these geometrical features elicit a subjective impression of transparency in the absence of the cues, such as X-junctions, that are normally required for perceptual transparency (3, 17–21) is a fascinating research question in its own right. Nevertheless, for the purposes of investigating how the visual system segments shapes by way of distinct causes, the artist’s technique of spatially alternating between overlying and underlying layers is problematic. For our purposes, we need images in which the two layers are superimposed, not side by side.

**Fig. 2.**
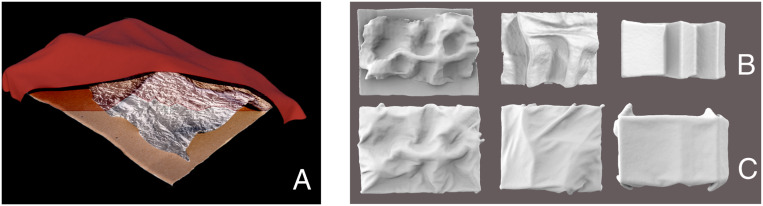
(*A*) Cross-section of 3D scan of stimulus A1, i.e., the first draping of base shape A. (*B*) Renderings of the three base shapes A to C (left to right), as presented in Experiment 2. (*C*) Renderings of example drapings of the three base objects, as used in Experiment 2 (from left to right: A2, B4, C1). For the complete stimulus set, see *SI Appendix*, Fig. S2.

To overcome these limitations, we created our own draped objects. The three underlying surfaces (“base shapes,” labeled A to C) consisted of unfamiliar reliefs, roughly 50 to 100 cm wide, made of cardboard and, in two of the three cases (base shapes A and B), chicken wire that was tightly coated in aluminum foil. These were digitized with a 3D scanner and then draped with cloth and scanned again so that we had ground-truth geometry for both underlying and overlying layers. Each base shape was draped multiple times (labeled with numbered values; e.g., stimulus A3 refers to the third draping of base shape A). The resulting 3D scans were rendered under naturalistic lighting (22) as ideal opaque Lambertian surfaces (Fig. 2). Rendering, rather than the use of photographs, enabled precise control of viewpoint and lighting, accurate coregistration of depth data with pixel data, and the use of ideal opaque surface reflectance to ensure the underlying surface was truly invisible to the observer. We presented these renderings to a new set of 69 participants in random order with similar instructions to those used with *The Veiled Virgin* sculpture (i.e., to mark ridges due to contact and fabric). For comparison, participants were also asked to mark ridges on images of the undraped underlying reliefs (36 participants did this before seeing the draped versions of the objects; the others did it afterward).

### Participants’ Judgments Are Highly Consistent.

**Fig. 3.**
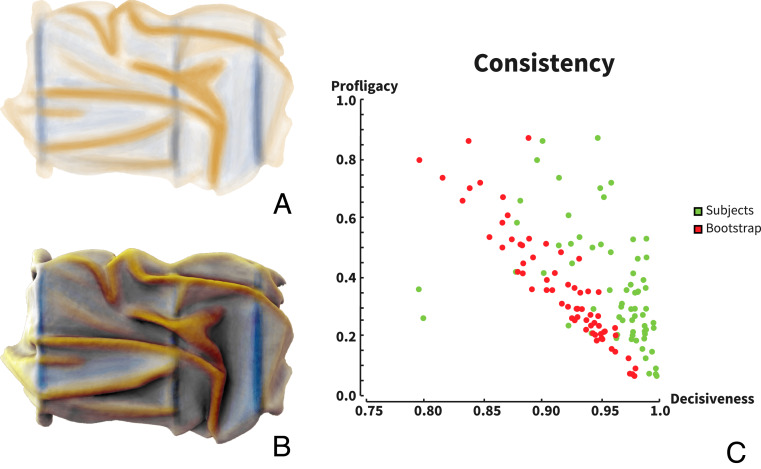
(*A*) Experiment 2: mean responses from 68 participants on stimulus C5. Blue indicates contact; yellow indicates fabric. (*B*) Data superimposed on the original image (see *SI Appendix*, Fig. S2 for all stimuli). (*C*) Profligacy and decisiveness of participants’ responses (see *Materials and Methods* for definitions). Green dots indicate responses for individual participants. Red dots indicate results of bootstrapping with random responses, statistically matched to individual participant data.

Mean responses across participants for one example draping is depicted in Fig. 3 (see *SI Appendix*, Fig. S2 for the complete set). We again evaluated the extent to which participants were internally consistent in attributing causes to each location by comparing the profligacy and decisiveness metrics with random responses. This analysis reveals that participants are significantly and substantially more decisive than would occur by chance. Participants’ markings were also highly consistent with one another; the mean correlation between maps produced by different observers for each stimulus was *r* = 0.52. We based our further analyses on the mean responses across participants (“mean maps”). As with the previous experiment, a within-subject paired comparison *t* test reveals that the observers’ distributions are significantly more decisive than their corresponding simulations: 66 of 68 participants showed significant differences between simulated and actual markings (*P* < 0.05), and 58 of 68 were significant with *P* < 0.01.

### Contact Responses Are Clustered by Base Shape.

**Fig. 4.**
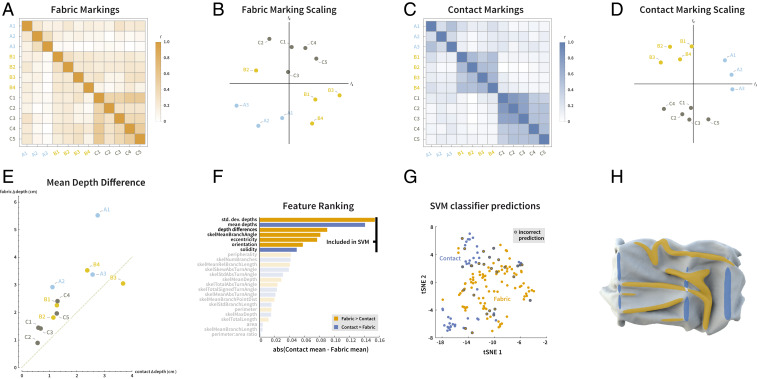
Label colors correspond to the three different underlying surfaces (cyan: A; yellow: B; green: C). (*A*) Correlations between the fabric maps for all stimuli revealing that participants’ responses were dominated by the differences between drapings. (*B*) Application of MDS to the correlation matrix reveals a disorderly arrangement in 2D, again reflecting differences in the shape of the fabric across stimuli. (*C*) Correlations between the contact maps for all stimuli, revealing greater similarities between stimuli that share the same base shapes. (*D*) Applying MDS to the correlation matrix reveals clear clustering in 2D MDS space. (*E*) Mean physical depth difference between textile and base shape for fabric and contact markings. Most points are above the diagonal, indicating larger depth offsets for fabric responses than for contact responses. (*F*) Ranking and selection of features in SVM classifier model: bars indicate magnitude of difference in mean of normalized feature values; color indicates sign (orange: fabric > contact; blue: contact > fabric). Transparency indicates features not used in SVM classifier. (*G*) SVM classifier predictions of causal assignment for all 154 segmented image regions. Coordinates are 2D tSNE visualization of each region in seven-dimensional (7D) feature space used for classification (orange: predicted fabric; blue: predicted contact; gray rings: incorrect predictions). (*H*) Predicted causal assignments of image regions for one stimulus (see *SI Appendix*, Fig. S2 for all stimuli) (orange: predicted fabric; blue: predicted contact).

It is also interesting to consider how accurately participants could identify the causes of features. We can use the fact that we created multiple drapings of each base shape to test whether participants could correctly distinguish between contact and fabric. If they could, then fabric markings for different drapings on the same base shape should be quite different from one another, while contact markings should be quite similar. To test this, we calculated the correlation between the mean maps for fabric and contact responses for each stimulus (Fig. 4). Applying multidimensional scaling (MDS) to the resulting similarity matrices reveals a disorderly arrangement for the fabric responses but a clear clustering by base shape for the contact settings. In other words, participants marked similar features when indicating the underlying surface, despite large variations in the visible shape, caused by the drapery. This strongly indicates that participants could indeed distinguish between features that were due to the base shape and those that were due to the textile’s own ripples and folds on top.

### Object-To-Textile Depth Differences for Fabric and Contact Markings.

As another test of accuracy, we took advantage of the fact that we had ground-truth depth data for both the overlying textile and the underlying surface. We computed for each pixel, the difference in 3D depth between the scans of the textile and base shape (“depth difference”) and compared these to participants’ responses. If participants perform the task accurately, we should expect the mean depth differences to be significantly smaller for contact than for fabric markings. Fig. 4 shows the mean depth difference within contact and fabric markings for each stimulus, weighted by the number of responses per pixel. All but one of the stimuli fall above the diagonal, indicating larger depth differences for the fabric than contact markings. This suggests that participants are broadly capable of separating shape features based on their causal origin.

### Predicting Causal Assignments.

Identifying which visual cues participants use to determine the causal origin of shape features is extremely challenging as the physics of textile is complex, and we carefully designed our stimuli so that the two causes led to similar geometrical structures. In a given stimulus (e.g., B3; *SI Appendix*, Fig. S2) fabric ridges might be smaller, flatter, and straighter than the contact features. However, in other stimuli (e.g., C5; Fig. 3), the tendency is reversed, precluding classification based any single simple cue. Nevertheless, we reasoned that the participants’ markings might be distinguished by a combination of multiple features, taking both the 2D and 3D properties into account.

To test this, we sought to predict the causal assignment of regions (i.e., whether participants assigned a given set of pixels to contact or fabric maps) by training a classifier based on 2D and 3D features derived from the ground-truth geometry and the participants’ markings. Specifically, we first thresholded and segmented the mean responses for each stimulus to identify regions that were most consistently marked across participants, yielding 154 regions across 12 stimuli. Then, for each region, we computed 24 different features describing the size, shape, orientation, and depth structure of the region (see *Materials and Methods* for the complete feature list). We ranked the features for informativeness by measuring the difference in mean of the distributions of normalized features values from contact and fabric regions (Fig. 4*F*). This revealed that 1) each feature in isolation is a relatively poor predictor of causal origin (the maximum difference was <16% of the total range of values); 2) the three most informative features (mean depth, SD of depth, and mean depth difference between overlying and underlying surface) were all related to the 3D structure, rather than 2D properties of the regions; and 3) contact markings were on average slightly closer to the observer, shallower, closer in depth to the base-shape, less elongated, more vertical, more compact, and had a skeletal structure with smaller branching angles (22, 23).

We then trained a support vector machine (SVM) classifier to predict the causal assignment of each region to either fabric or contact, based on the feature values of the top seven most informative features (*Materials and Methods*). The regions in each stimulus were predicted from SVMs trained exclusively on the other 11 stimuli, yielding 82.5% correct classifications across all 154 regions. Fig. 4*G* shows a t-distributed stochastic neighbor embedding (tSNE) visualization of the contact and fabric regions in a 2D projection of the feature space, with circles indicating misclassifications. We can project the predicted labeled regions back onto the stimuli, as in Fig. 4*H* for stimulus C5 (see *SI Appendix*, Fig. S2 for the complete set). In most cases, the predicted causal assignments closely resemble those in the raw data, indicating that a combination of relatively simple 2D and 3D features are sufficient to capture the differences between contact and fabric markings.

This analysis is not a process model of human visual causal inference and does not reveal the specific cues that the visual system uses. Indeed, the 3D features are not computed from the image but derived from the ground-truth geometry. The selection of which features to consider was based on loose phenomenological intuition rather than derived from first principles. Nevertheless, the fact that the 3D properties were the most informative of the features we considered reinforces the fact that the causal attribution likely involves rich representations of the surface geometry, rather than simple 2D geometrical properties. This leaves open the possibility that participants might explicitly estimate the depths of both layers or may at least be able to interpolate depths of the underlying surface when presented with sparse indications of its relief at the regions of contact. To test these possibilities, we performed a third experiment.

### Experiment 3: Depth Reconstructions of Superimposed Surface Layers.

Although participants reported finding the painting task highly intuitive, on its own it does not indicate whether their representation of the draped objects was layered, with multiple causes superimposed along a given line of sight. The findings show that participants can categorize directly visible features by their causes, but it remains unclear whether observers perceive one surface hidden behind another. We reasoned that if participants understand the composite shape as multilayered, they should, at least to some extent, be able to provide independent estimates of both surface reliefs simultaneously. Even if they cannot directly perceive multiple depths at each point in the image, the combination of causal attribution and depth-interpolation mechanisms (24–33) might enable them to report not only the shape of the overlying cloth but also (at least approximately) the shape of the underlying surface, even at locations where the surface is not in contact with the textile.

To test this, we performed an additional experiment in which 12 experienced observers were asked to draw the depth profile shape of cross-sections through the draped stimuli for both the overlying textile surface and the underlying base shape simultaneously. The experiment was again conducted via a tablet computer, and on each trial, participants viewed one of the renderings with a given raster line highlighted. Their task was to report the depth relief of both the overlying and underlying surfaces along that raster line via a 2D contour that they could draw and manipulate. Below the stimulus, a region was presented of the same width as the stimulus, within which they could create and adjust contours representing the two surface reliefs (fabric and base shape). Rather than freely drawing the shapes, which participants find demanding and unintuitive, the contours consisted of piecewise straight lines passing between control points, which could be freely added, deleted, or moved by the participant.

**Fig. 5.**
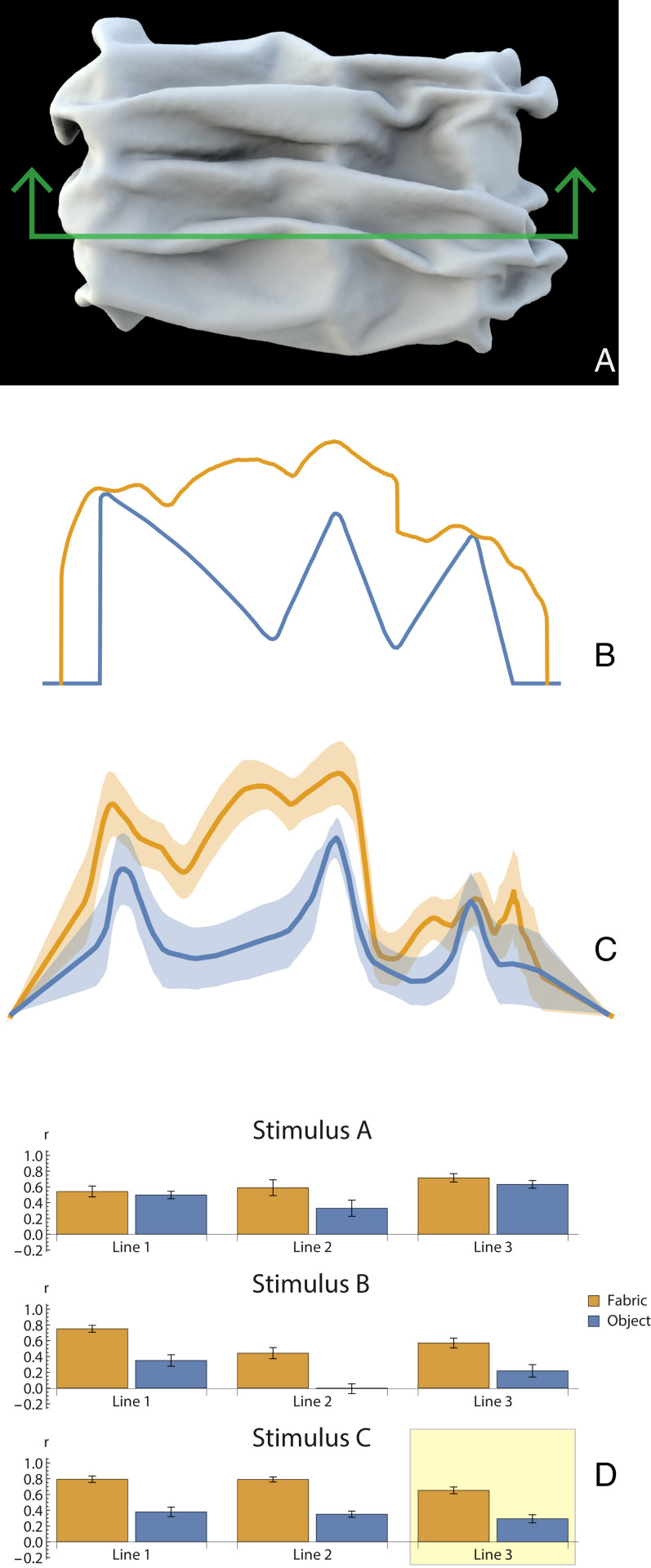
(*A*) Experiment 3: stimulus C4, with green raster line indicating one cross section whose depth profile participants were asked to estimate. (*B*) Ground-truth depths along raster line (blue: underlying base shape; orange: overlying textile). (*C*) Mean responses across 12 participants, for the same raster line (blue: estimated base shape depths; orange: estimated textile depths). (*D*) Correlations between all ground-truth depths and participants’ responses for all nine raster lines (blue: base shape; yellow: fabric). Example stimulus is shown highlighted in yellow. For all stimuli, see *SI Appendix*, Fig. S3.

To combine data across participants, we normalized the responses for each raster line independently to the range of 0 to 1 and took the mean. An example mean response is shown in Fig. 5 for one raster line (see *SI Appendix*, Fig. S3 for the complete dataset). For comparison, Fig. 5 shows the true depth profiles of the two layers for the corresponding raster line. To evaluate performance, we computed the correlations between participants’ responses and the true depth profiles for each of the two surfaces along each raster line (Fig. 5*D*). The correlations were significant in all cases. As is to be expected, in all cases, the depth-profile responses for the overlying fabric were significantly better correlated with the ground truth than for the underlying surface. This presumably reflects the fact that the overlying surface is in plain view, and thus conventional shape from shading mechanisms can deliver information about surface relief, whereas the structure of the hidden surface must be inferred from the sparse regions of contact between textile and surface.

It is well known that this kind of task yields substantial metric errors in reporting the depths of surfaces (34–36). This is reflected in the errors in responses for the overlying surface, in which no perceptual scission was required; participants simply had to report the directly visible depth relief, as in previous studies. Despite this, the results suggest that, at least qualitatively, participants can report the depth profiles of both visible and hidden surfaces. This suggests that participants do understand the composite shape as multiple superimposed surfaces and, when asked to, can separately report the depth profiles of these layers. The fidelity of the reconstructions unlikely suffices for a straightforward thresholding on estimated depth values as the basis for painting responses in Experiments 1 and 2. We suggest, that the processes of estimating depth profiles of both layers and of assigning causes (fabric vs. contact) to visible surface ridges are related but not one and the same. Specifically, we suggest that having identified distinct causes, the depth reconstruction for the underlying surfaces relies on mechanisms that group and interpolate the sparse depth signals visible at contact locations.

## General Discussion

Taken together, our results suggest that the representation of 3D shape in the human visual system is richer than a map of local surface properties, such as depth, orientation, or curvature (37–43). Instead, shape perception also supports inferences about other aspects of objects, including their material properties and causal origin. When an object is hidden behind opaque cloth, it cannot be seen directly, yet it visibly influences the shape of the overlying textile layer. We find that observers can distinguish between shape features that are due primarily to the hidden object and those that result from the textile organizing itself into creases, folds and other structures. We call this the “*Veiled Virgin* effect.” It is an important visual inference because, for example, in order to grasp and handle an object successfully, we need to know its compliance. Unsupported cloth tends to yield and change shape under the slightest of touches, whereas when the cloth is tightly bound around another object, we should anticipate encountering considerably larger normal forces. However, the inference of the causal origin of shape features and the structure of hidden objects is extremely challenging (6–16, 44).

Yildrim et al. (44) found that participants were good at identifying which of several candidate objects was hidden under simulated draped textile. They developed a computational model based on approximate physical simulation and showed that it outperformed a conventional deep neural network retrained to recognize the hidden objects. On this basis, they suggested that an internal “physics engine” (45, 46) may play a central role in such tasks. However, it remains unclear if human observers can or even need to perform an explicit mental physical simulation in order to identify the causal origin of specific shape features, as the participants in our experiments did.

There is also a close connection between our findings and those of amodal completion—the subjective impression that a surface continues behind an occluder that renders part of it invisible (24, 28, 29, 47–49). In particular, it has been noted that certain conditions elicit volume completion (25–27), such that hidden portions of objects seem to fill volumes in unseen space. This can occur even in the absence of local occlusion cues (e.g., T-junctions and “relatable” contours; ref. 24) that are traditionally associated with amodal completion. However, there is one important difference between our findings and amodal completion: in the *Veiled Virgin* effect, the “completed” object is entirely hidden from view. Instead of visible inducers initiating an interpolation of surface structure across gaps in the sensory signal, here, the only way to infer the presence of the hidden surface is by detecting and interpreting the effects it has on the shape of another surface. However, depth interpolation surely plays a central role in the percept. In Experiment 3, when asked explicitly to report the depth relief of the invisible surface, participants likely relied primarily on interpolating between the depths of the visible contact regions. The details of these depth-interpolation processes should be investigated in more detail in future studies.

We suggest that causal attribution is achieved, at least in part, through recognition mechanisms. That is, because we are familiar with cloth, we can identify tell-tale signatures of its typical behavior when it freely self-organizes under ambient ecological effects, such as gravity, while deviations from typical shape features are likely caused by a hidden object. Drapery exhibits distinctive geometrical features (50–52), and even though an observer is unlikely to have not seen a given specific configuration of folds or creases before, as an ensemble, they allow the observer to recognize cloth. Indeed, recent studies indicate that observers can use shape, motion, and the optical properties of textiles to infer their mechanical characteristics (53–57). We suggest that features of the shape of the cloth that do not conform to familiar types of ridge and fold structure could thereby be identified as outliers that likely have a separate cause, i.e., a hidden object that interrupts the cloth's natural flow. In particular, when freely draping, materials that resist stretching tend to organize themselves into shapes with (piecewise) low Gaussian curvature as these are lower energy than doubly curved surfaces. Thus, extended regions of double curvature (e.g., hills, dales, and saddles) are likely indicators of a hidden object perturbing the cloth (e.g., when the cloth is pulled taut over another object). Moreover, depending on the density, stiffness, and other properties of the material, it will tend to exhibit certain curvatures or frequencies of periodic structure (e.g., the ripples in curtains). Gross deviations from these statistics, such as sharp corners, could also be an important indicator of contact between the cloth and a hidden object. It is important to note, however, that the subjective impression of something hard hidden behind something soft is much more general than textile drapery. For example, the protrusions caused by the knuckles, pelvis, or ribs under the skin clearly indicate the presence of the underlying bone (we expect the region to feel firm if prodded), even though there are none of the geometrical features that are typical of drapery (58, 59). Thus, detecting hidden objects is clearly more complex than just the detection of cloth features and deviations therefrom.

Indeed, the cues underlying such inferences need substantial additional research. We were able to build a classifier that could predict participant judgments surprisingly well based on some very simple 2D and 3D measurements. The fact that features selected in an hoc manner performed well suggests that fabric and contact regions in our stimulus set do display many differences. However, it is unlikely that the classifier would generalize well to a broader range of stimuli with different base shapes and textile properties, and it is highly unlikely that the visual system uses these exact image and depth estimate measurements to identify the causes of shape features. Identifying the geometrical features predicted by physical models of textiles in images is highly challenging. Learning-based approaches driven by much larger training sets may yield some useful insights into more general cues that humans might rely on.

The *Veiled Virgin* effect is also closely related to the perception of transparency (3, 4, 17–21). One of the most striking aspects of *The Veiled Virgin* sculpture is the impression that the face is seen through a highly transparent diaphanous veil. However, it is important to note that the perception of transparency itself is orthogonal to the perception of multiple causal contributions to observed shape. Our unfamiliar stimuli are rendered as perfectly opaque and elicit no impression of transparency. Nevertheless, what transparency and the *Veiled Virgin* effect share is the decomposition of sensory signals into multiple causal layers (also known as scission; refs. 3, 7, 17, and 18). In perceptual transparency, a single retinal luminance or color is parsed into two subjective layers: a background of a certain reflectance seen through a transparent layer with a different reflectance and opacity. How the visual system achieves this is still not fully understood, although it involves comparing contrasts seen through the transparent layer with those from the background seen in plain view. In contrast, in the *Veiled Virgin* effect, to the extent that there is any scission into causal layers, this is not a separation of luminance or color but rather of geometry. This seems like a much higher-level process. At some level, observers certainly “understand” that the stimulus consists of multiple layers, even if they cannot explicitly “perceive” both layers directly. Presumably, shape can only be segmented into causes once the 3D relief has been estimated. However, the interaction between causal inferences, depth interpolation, physical simulation, and mental imagery remains poorly understood. Certainly, the question of how purely geometrical features in the *Veiled Virgin* sculpture can elicit a perception of transparency in the absence of any of the cues or conditions that are typically associated with transparency perception (e.g., X-junctions, distortions of a background pattern; ref. 60) is perhaps as beguiling as the sculpture itself.

## Materials and Methods

All experiments were conducted in accordance with the Declaration of Helsinki, and procedures were approved by the local ethics committees of the Departments of Psychology at Giessen and Skidmore College. Prior to the start of each experiment, participants were informed that they could terminate the experiment at any time and gave consent for their data to be published.

### Experiment 1: Painting Task on *The Veiled Virgin* Sculpture.

Two high-quality, high-resolution images of *The Veiled Virgin* were supplied by the Presentation Sisters of Newfoundland Canada—home of the sculpture. The images, depicting the sculpture from two different points of view, were subject to cropping to highlight features at different scales. In all, each image was presented as a whole-sculpture, full-face, nose-mouth, and eye detail, resulting in eight total stimuli (*SI Appendix*, Fig. S1). Sixty subjects were instructed to indicate areas on the sculpture where the fabric veil was perceived as making contact with the underlying structure (i.e., the Virgin) and where the fabric was seen as being separate and elevated away from the underlying structure (fabric). Responses were collected using a bespoke iPad & Apple Pencil application, written in Python (61), that provides a traditional digital-painting interface. After brief instructions as to the task and application interface, subjects were given the mostly unconstrained ability to mark each of the two conditions—contact or fabric—on each image. To allow measurement of perceptual ambiguity, the application only displayed the subjects’ markings for a single condition at a time. This permitted marking the same location as being seen as one of the interpretations, both simultaneously or neither. Finally, the application provided for the unlimited ability to erase and toggle between the two marking conditions.

Markings for each observer were analyzed by calculating two measures from the fabric and contact stroke maps—namely profligacy and decisiveness. Profligacy is defined as the proportion of possible pixels marked and is calculated as the union of the fabric and contact maps:

$$\text{P}=\text{N}\left({\text{I}}_{\text{fabric}}\cup {\text{I}}_{\text{contact}}\right)/{\text{N}}_{\text{max}},$$

where N represents the total number of pixels marked, and N_max_ is the total number of pixels contained within the boundary contour of the stimulus image. The binary stroke maps (I_fabric_, I_contact_) contain the observers’ indications of fabric ridges and fabric contact, respectively. Profligacy yields a measurement of the overall assignment of causal information, regardless of its source. Profligacy scores near 0 indicate very little of the stimulus was marked as containing either fabric or contact information, while scores near 1 suggest that most pixels were judged as being part of the fabric, contact, or both.

Decisiveness indicates how unique the fabric and contact assignments are. It is computed as the inverse of the intersection of the two stroke maps:

$$\text{D}=\text{1}-\text{N}\left({\text{I}}_{\text{fabric}}\cap {\text{I}}_{\text{contact}}\right)/{\text{N}}_{\text{max}}.$$

Low decisiveness corresponds to higher ambiguity in observers’ markings and vice versa.

### Experiment 2: Painting Task on Unfamiliar Objects.

Three physical, 3D surface reliefs, measuring ∼1.0 × 0.75 × 0.25 m, were sculpted from cardboard and chicken-wire using traditional maquette construction techniques. The resulting forms provide varying amounts of geometric information ranging from simple, linear structures to curvilinear ridges and valleys and peak and dimple locations. Each surface was digitized using an iPad-based 3D laser scanning device (62) and software customized for this specific scanning task. The resulting geometry acts as the ground truth of underlying structure—one that is not directly available from *The Veiled Virgin* sculpture since the veil cloth and underlying geometry of the face are physically integrated and inseparable in the sculpted depiction. The complete set of stimuli is reproduced in *SI Appendix*, Fig. S2 and is available for download from Zenodo under DOI: 10.5281/zenodo.3766182.

Each underlying ground truth was then draped using a midgray batiste cloth in an assortment of configurations. These draped surfaces were then digitized using the same method as the underlying surfaces. Finally, the resulting surfaces were rendered as ideal Lambertian surfaces using Radiance (63). Rendering enabled precise control of viewpoint and lighting, coregistration of depth data with pixel data, and the use of ideal opaque surface reflectance to ensure the underlying surface was truly invisible. This set of images acted as the stimuli for an experiment using the same method, software, and analysis as Experiment 1. In addition, as we have the exact measured geometry underlying the images presented to participants, we could also test the extent to which participant’s judgments varied with the depth offset between underlying relief and overlying textile.

### SVM Classifier.

Analyses were conducted in Matlab. Mean contact and fabric maps for each stimulus were preprocessed and segmented to identify regions consistently labeled by participants as follows: mean values were Gaussian blurred (sigma: 10 pixels), squared (to emphasize higher values), normalized to the 0 to 1 range, and then binarized with a threshold of 0.3. The “imopen” function was applied with a one-pixel disk structured element to remove tiny regions. The resulting images were then segmented using “bwconncomp,” yielding a total of 154 connected component regions across all 24 images. From each region, the 24 candidate features in Table 1 were computed using a combination of “regionprops” and Bayesian skeleton features using ShapeToolbox1 by Feldman and Singh (23) (https://ruccs.rutgers.edu/images/ShapeToolbox1.0.zip). Code for extracting the skeleton summary statistics reported by Wilder et al. (64) was developed by Yaniv Morgenstern.

**Table 1.** Candidate features computed from image regions

Feature name	Description	Source
Area	Area of region in pixels	Regionprops: Area
Orientation*	Orientation of best-fitting ellipse in degrees	Regionprops: Orientation
Eccentricity*	Ratio of principal axes of best fitting ellipse	Regionprops: Eccentricity
Perimeter	Perimeter of region in pixels	Regionprops: Perimeter
Solidity*	Ratio of filled area to area of convex hull of region	Regionprops: Solidity
perimAreaRatio	Ratio of perimeter to area of region	Derived from regionprops: Perimeter and Area
Peripherality	Euclidean distance of region’s center of mass from center of image	Derived from regionprops: “Centroid”
meanDepths*	Mean depth values of surface within region	Regionprops: “MeanIntensity”
stdDepths*	SD of depth values of surface within region	Derived from regionprops: “PixelValue”
depthDifferences*	Mean value of depth difference between overlying and underlying surfaces within region	Regionprops: “MeanIntensity”
skelNumBranches	Number of branches in skeleton of region	Bayesian Skeleton of region computed using ShapeToolbox1 (23); many of the features are described in more detail by Wilder et al. (64)
skelMaxDepth	Maximum graph depth of branches in skeleton	
skelMeanDepth	Mean graph depth of branches in skeleton	
skelMeanBranchAngle*	Mean angle of branch from parent	
skelMeanBranchPointDist	Mean distance along parent at which branch emerges	
skelMeanRelBranchLength	Mean length of branch relative to total length of skeleton	
skelTotalAbsTurnAngle	Total absolute turning angle of skeleton	
skelTotalSignedTurnAngle	Total signed turning angle of skeleton	
skelMeanAbsTurnAngle	Mean absolute turning angle along skeleton branches	
skelStdAbsTurnAngle	SD of absolute turning angles along skeleton branches	
skelSkewAbsTurnAngle	Skewness of absolute turning angles along branches	
skelTotalLength	Total length of skeleton	
skelMeanBranchLength	Mean branch length of skeleton	
skelStdBranchLength	SD of branch lengths along skeleton	

* Only those indicated with an asterisk were used in the SVM classifier.

Where features returned "not a number" (NaN) for a given region, these were replaced with the mean value across all other (real-valued) regions. For each feature, the range of values across all regions was normalized 0 to 1. The top seven features in terms of absolute difference in the mean for distinguishing the contact and fabric distributions were used as the basis for training the SVM classifier using “fitcsvm.” For each stimulus, a separate SVM classifier was trained using the regions from the other 11 stimuli. Training labels were binary values indicating whether a region came from a contact or fabric map. For each SVM, Bayesian search (50 iterations) was used to identify values of the following hyperparameters that yielded best cross-validated performance on each training set: “BoxConstraint,” “KernelScale,” “KernelFunction,” “PolynomialOrder,” and “Standardize.” These typically yielded second- or fourth-order polynomial kernels (thus, KernelScale was typically undefined). BoxConstraint varied widely (mean: 50.25; range: 0.0014 to 548.82), and Standardize was “false” for seven and “true” for five of the runs. Inferred causal attributions for the regions in each stimulus were then derived using the “predict” function from the SVM trained on the other stimuli. The tSNE visualization in Fig. 4*G* used the default parameters.

### Experiment 3: Two-Layer Depth Profiles on Unfamiliar Objects.

Stimuli consisted of three of the rendered objects from Experiment 2 (base shape 1, draping 10; base shape 2, draping 8; base shape 3, draping 4). For each of these, three horizontal raster lines were selected in which there was substantial variation in the distance between the underlying and overlying surfaces (*SI Appendix*, Fig. S3). Stimuli were presented on a 12-inch iPad Pro, running a bespoke Python program (61) and responses made using an Apple Pencil. Twelve experienced observers participated (unpaid). Observers were unfamiliar with the stimuli and the purpose of the study. On each trial, the rendering was presented, and a single horizontal raster line was highlighted in blue. Directly below the stimulus was a response region with exactly the same horizontal extent as the image. The participants’ task was to report the perceived depth relief of the surface along the raster line by drawing a graph of depths within in the response region. As free drawing is challenging, we used an interface whereby participants added, moved, and (optionally) deleted control points, to shape a piecewise linear contour representing the relief function. Control points could be moved in both *x* and *y* directions. Importantly, participants were instructed to provide two contours for each raster: one representing the relief of the overlying textile (seen directly), and the other representing the relief of the hidden underling surface. Participants could toggle which of the two contours was currently editable via buttons labeled “drapery” and “object” in the interface, but both contours were visible throughout each trial. Participants were instructed to use the *y* axis to represent height above the ground plane, such that higher *y* values indicated surface positions closer in depth to the observers. They were also instructed to anticipate the total range of responses across both contours to ensure they did not run out of space within the response area. Data from each raster line were normalized to the range of 0 to 1, preserving the relative heights of the drapery and object responses. An example of the interface is shown in *SI Appendix*, Fig. S4.

### Data Availability.

Raw data have been deposited in Zenodo under DOI: 10.5281/zenodo.3766182.

## Supplementary Material

Supplementary File
