# Supplementary material for: The Veiled Virgin illustrates visual segmentation of shape by cause
Source: Proc Natl Acad Sci U S A. 2020 May 15;117(21):11735–43. doi: 10.1073/pnas.1917565117 (PMC7260992; doi:10.1073/pnas.1917565117)
Supplement: Supplementary File [file pnas.1917565117.sapp.pdf]

PNAS
www.pnas.org

Supplementary Information for

The *Veiled Virgin* illustrates visual segmentation of shape by cause

Flip Phillips[1,2,*] and Roland W. Fleming[3,4,*]

[1] Department of Motion Picture Science, Rochester Institute of Technology, USA

[2] Department of Psychology and Neuroscience, Skidmore College, USA

[3] Department of Psychology, Justus-Liebig-University Giessen, Germany

[4] Center for Mind, Brain and Behavior, Marburg and Giessen Universities, Germany

*** Equal Authorship**

Corresponding author:

Flip Phillips

Department of Motion Picture Science, MAGIC Center, Rochester Institute of Technology, Rochester, NY, 14623-5608, USA

Tel: +1 585 475-4999

flip.phillips@rit.edu

This PDF file includes:

Figures S1 to S4

www.pnas.org/cgi/doi/10.1073/pnas.1917565117

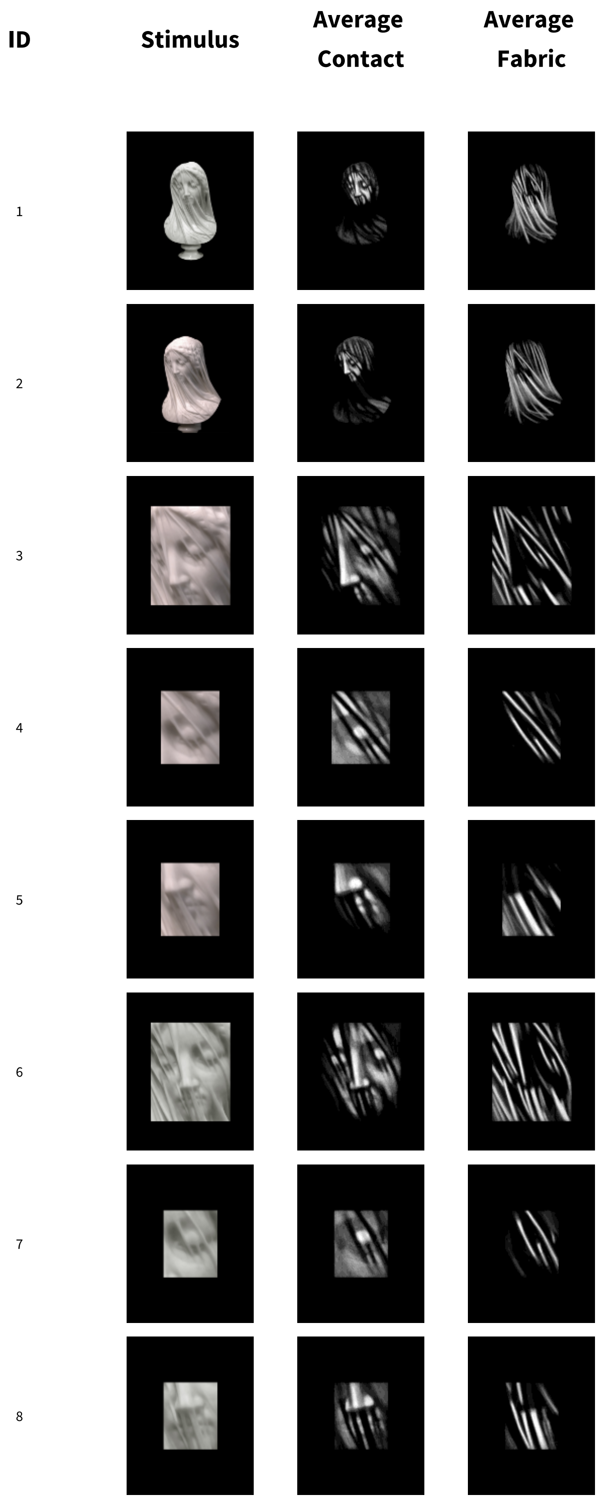

Fig. S1. All stimuli used in Experiment 1 with averaged responses for each.

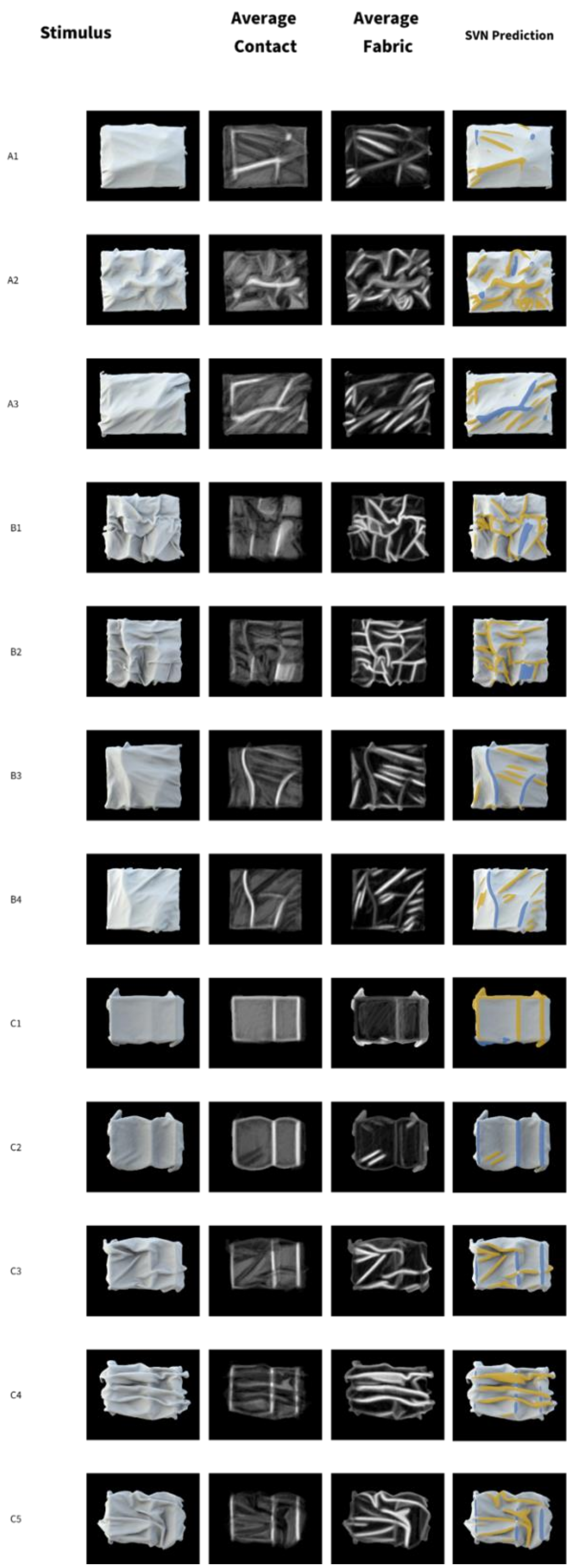

Fig. S2. All stimuli used in Experiment 2 with averaged responses and model predictions.

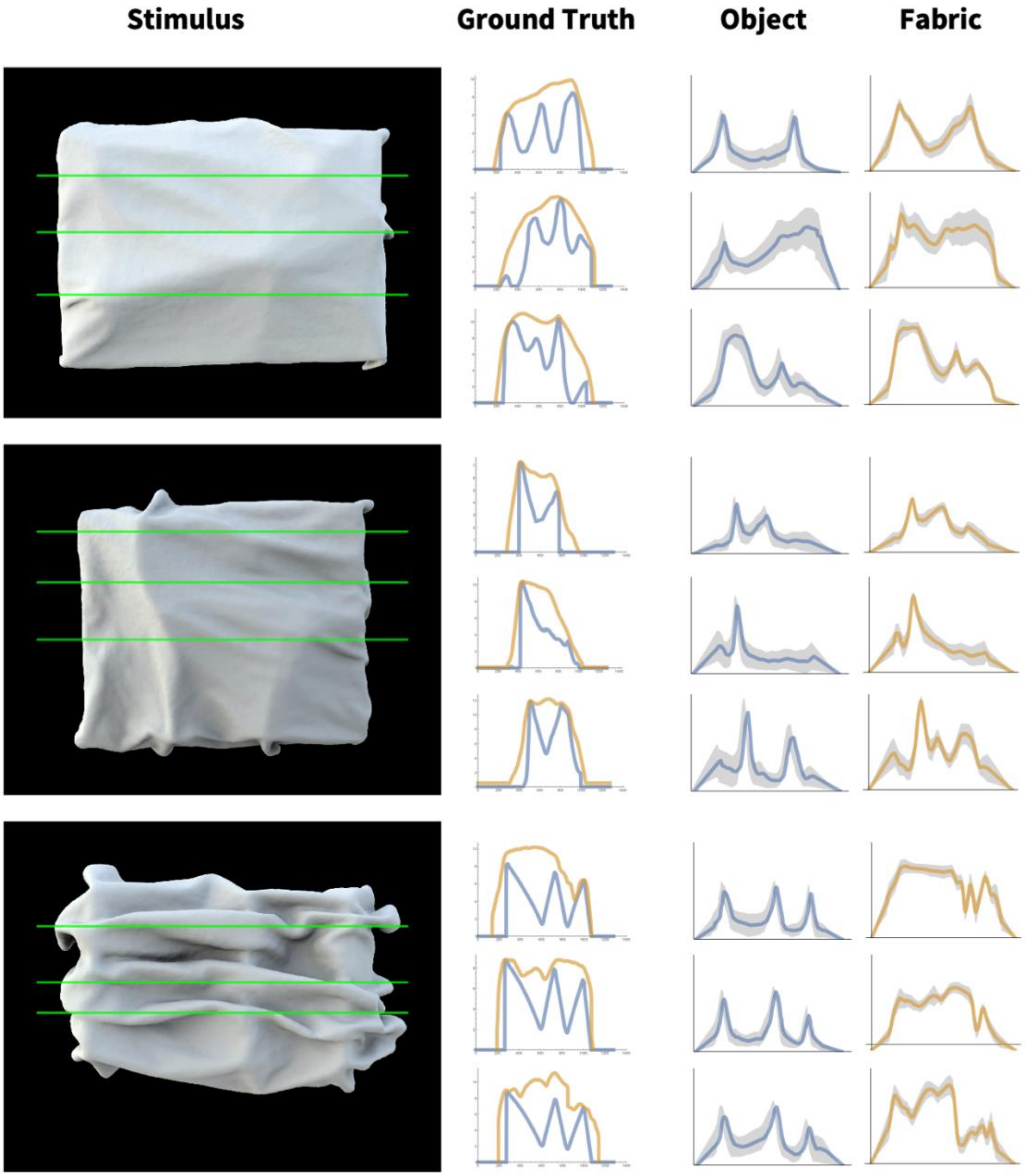

Fig. S3. All stimuli used in Experiment 3 with ground-truth profiles and mean responses and 95% C.I.s.

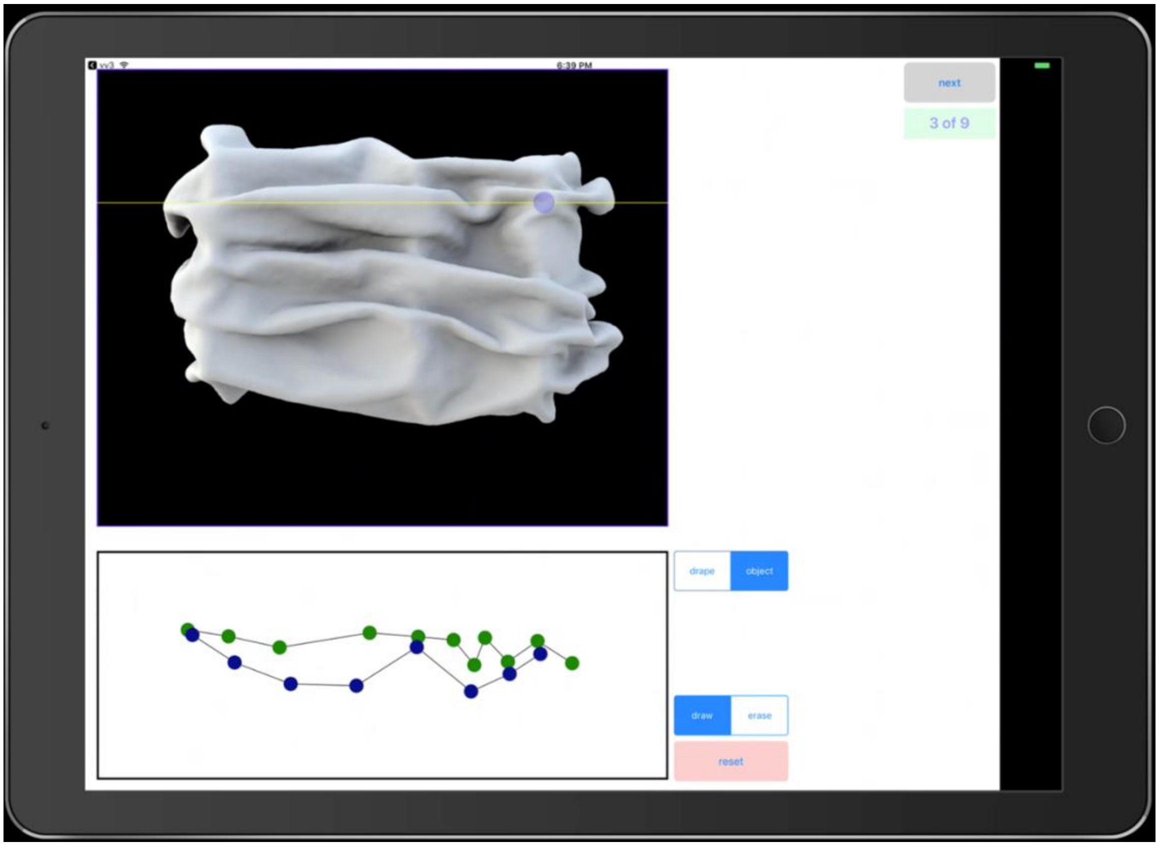

Fig. S4. Illustration of example session from Experiment 3.
